# Ongoing transmission of onchocerciasis in the Pru District of Ghana after two decades of mass drug administration with ivermectin and comparative identification of members of the *Simulium damnosum* complex using cytological and morphological techniques

**DOI:** 10.1186/s13071-024-06333-2

**Published:** 2024-09-18

**Authors:** Friday Maduka Chikezie, Francis Balunnaa Dhari Veriegh, Samuel Armoo, Daniel Adjei Boakye, Mark Taylor, Mike Yaw Osei-Atweneboana

**Affiliations:** 1 Department of Animal and Environmental Biology, University of Uyo, Uyo, Akwa Ibom State Nigeria; 2 African Regional Postgraduate Program in Insect Science, University of Ghana, Legon, Accra, Ghana; 3 Biomedical and Public Health Research Unit, Water Research Institute, Council for Scientific and Industrial Research (CSIR), Accra, Ghana; 4 University of Energy and Natural Resources, Sunyani, Ghana; 5 The END (Ending Neglected Diseases) Fund, New York, NY USA; 6 Noguchi Memorial Institute for Medical Research, University of Ghana, Legon, Accra, Ghana; 7 Liverpool School of Tropical Medicine, Liverpool, UK; 8 College of Science and Technology, Council for Scientific and Industrial Research (CSIR), Accra, Ghana

**Keywords:** Onchocerciasis, *Onchocerca volvulus*, *Simulium damnosum*, Transmission, Black flies, Cytotaxanomy

## Abstract

**Background:**

Human onchocerciasis remains a public health problem in Ghana. Mass drug administration (MDA) with ivermectin (IVM) has reduced disease morbidity and prevalence, but the transmission of onchocerciasis remains ongoing in several endemic foci. We investigated parasite transmission in some endemic communities in Ghana that had received > 18 rounds of annual MDA with IVM and determined the species composition of black fly (*Simulium damnosum*) vectors in these areas.

**Methods:**

Adult female black flies were collected using human landing catches and identified as either forest or savanna species using morpho-taxonomic keys. The adult flies underwent dissection to determine their parity and detect any *O. volvulus* larvae, followed by the calculation of entomological indices. *Simulium damnosum* s.l. larvae were collected and preserved in freshly prepared Carnoy’s fixative and were later used for cytotaxonomic studies.

**Results:**

A total of 9,983 adult flies were caught: 6,569 and 3,414 in the rainy and dry seasons respectively. Black fly biting activities over the study period showed bimodal or trimodal patterns. The highest monthly biting rate (MBR) of 10,578.75 bites/person/month was recorded in July in Beposo, while the highest monthly transmission potential of 100.69 infective bites/person/month was recorded in Asubende in August. Morphological analysis of 2,032 flies showed that 99.8% (2,028) of the flies were savanna species, with only 4 (0.2%) adult flies being of the forest species. Cytogenetic studies on 114 black fly larvae revealed three cytospecies (*Simulium damnosum* s.s., *S. sirbanum* and *S. sanctipauli*) in the study area.

**Conclusions:**

The present studies confirmed an ongoing transmission of onchocerciasis in the study communities except Abua-1. It also provides further information on biting behaviors and onchocerciasis transmission indices in the study communities. Further, our data confirmed the savanna species (*S. damnosum* s.s. and *S. sirbanum*) of the *S.* *damnosum* s.l. to be the major vectors of onchocerciasis in the study areas, with only an occasional influx of forest cytotypes.

**Graphical Abstract:**

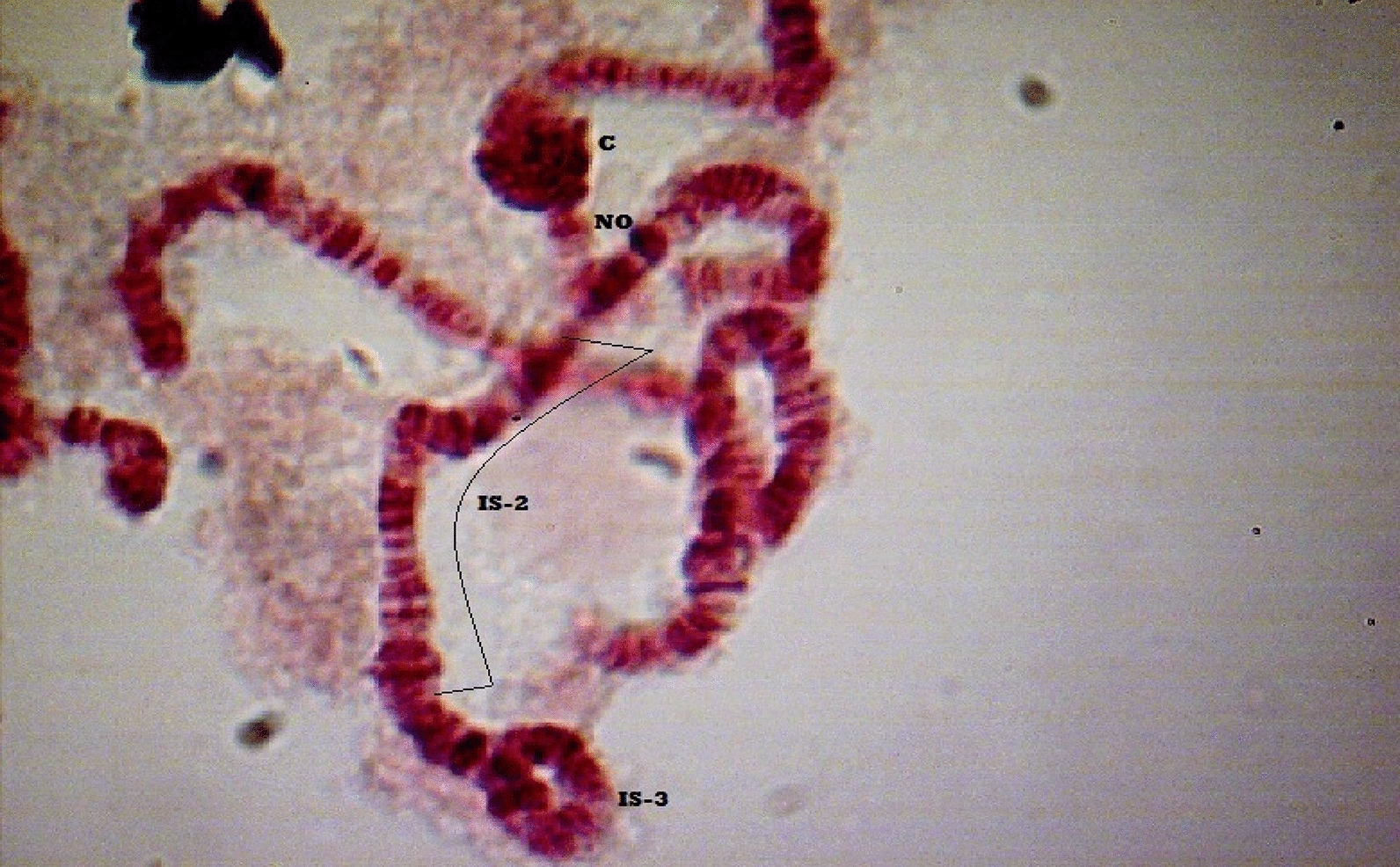

**Supplementary Information:**

The online version contains supplementary material available at 10.1186/s13071-024-06333-2.

## Background

Onchocerciasis is the second leading cause of infectious blindness in the world, only preceded by blinding trachoma [1–3]. The disease is a major obstacle to socio-economic development in Africa and other parts of the world [4, 5], being endemic in 30 countries in Africa, one country (Yemen) in the Arabian Peninsula and a few foci in the Americas [3, 6]. The adult stage of *Onchocerca volvulus*, the causative agent of the disease, have an average lifespan of 10 years and are found in the subcutaneous tissues of the host [7]. The adult female worms produce millions of microfilariae that migrate to the skin and eyes, where they cause dermal [7] and ocular [3] pathologies associated with the disease. Microfilariae in the skin are ingested during blood meals by adult female black fly vectors belonging to the *Simulium damnosum* complex, and later transmitted to human hosts as infective L_3_ larvae during another blood meals [8].

In West Africa, nine members of the *S. damnosum* species complex have been identified: *S. damnosum* s.s., *S. dieguerense*, *S. konkourense*, *S. leonense*, *S. sanctipauli*, *S. sirbanum*, *S. soubrense*, *S. squamosum* and *S. yahense* [9–12]. In Ghana, six members of this complex have been reported, and these are: *S. damnosum* s.s., *S. sirbanum*, *S. sanctipauli*, *S. soubrense*, *S. squamosum* and *S. yahense* [13, 14]. Several subcomplexes make up the *S. damnosum* complex, including the *S*. *damnosum* subcomplex, *S*. *squamosum* subcomplex, *S*. *sanctipauli* subcomplex (all of which are common in Ghana) and the *S*. *kibwezi* subcomplex, *S*. s*anje* subcomplex and *S*. *ketaketa* subcomplex (which are alien to the Ghanaian ecosystem).

The distribution of members of the *S*. *damnosum* complex in West Africa is reportedly associated with the savanna-forest dichotomic characteristics of the prevailing bioclimatic zones as well as with the river system types in West Africa. All black fly species described in West Africa to date are vectors of *O*. *volvulus*, albeit to varying degrees of vectorial capacity. For example, transmission of *O*. *volvulus* is maintained by members of* S. damnosum* s.l. (*S. damnosum* s.l.) in West Africa, by members of the *S. neavei* group in East and Central Africa and in the Americas by vectors belonging to six distinct species complexes [12, 15–17].

In West Africa, the epidemiological patterns are determined predominantly by the existence of two different strains of the *Onchocerca* parasite in which savanna and forest species of *Simulium* transmit their ecologically co-adapted savanna and forest strains of the *Onchocerca* worms respectively, even though this assertion has been strongly debated [16, 18, 19]. Thus the savanna cytospecies, *S. damnosum* s.s. and *S. sirbanum* will transmit the more pathologic savanna strain of *O*. *volvulus* while *S. sanctipauli*, the Beffa form of *S. soubrense*, *S. squamosum* (of which C and E forms exist) and *S. yahense* transmit the benign forest strain of *O. volvulus* [14]. Given the complex species structure within the genus *Simulium*, their different vectorial capacities, the unique associations with different geographical and ecological settings and the important role of vectors in maintaining disease transmission, it has become crucial to develop field tools for easily and accurately assigning species to their appropriate taxonomic units.

Since the work of Theobald [20] and Blacklock [21] in describing and incriminating onchocerciasis vectors respectively, there have been continual efforts to develop more robust morphometric tools for easy and accurate identification of adult members of the Simuliidae. In  recent times, however, there have been some challenges with using only the morphological identification technique [22, 23]. For example, the West African *S. damnosum* complex can be readily recognized by morphological characters at the larval and pupal stages but not at adult stage, but in Guatemala, adult females of *S. ochraceum*, *S. metallicum* and *S. callidum* can easily be distinguished even by color differences, whereas their immature stages are more difficult to separate morphologically [22]. Other challenges include the enigmatic nature of the constituent species; differences in behavior and ecological requirements; variations in habitat preferences; and genetic introgression [23]. Reliable identification of the members of the *S. damnosum* complex now requires a combination of both morpho-taxonomy and other tools [24]. Larval cytotaxonomy have been found to be a more reliable technique for vector species discrimination. It clarifies and widens our knowledge of the systematics [11, 25–29] and evolutionary biology [30–32] of the Simuliidae using micro-morphological features of the giant polytene chromosomes of late-stage larvae. It utilizes differences in banding patterns and inversions of polytene chromosomes in providing sufficient inter- and intra-specific variations within and among fly populations [33–37].

In this study, we sought to assess the status of *O*. *volvulus* transmission in the study communities and explore the the black fly species composition sustaining transmission of onchocerciasis in the study areas. Considering that the disease is endemic in nine of the ten regions of the country during the study period, with an at-risk population of 3.2 million people [38], this study is crucial. Moreso, because all our study communities had undergone annual IVM MDA for >18 years and are now under the biannual IVM treatment strategy.

## Methods

### Study areas

**Fig. 1:**
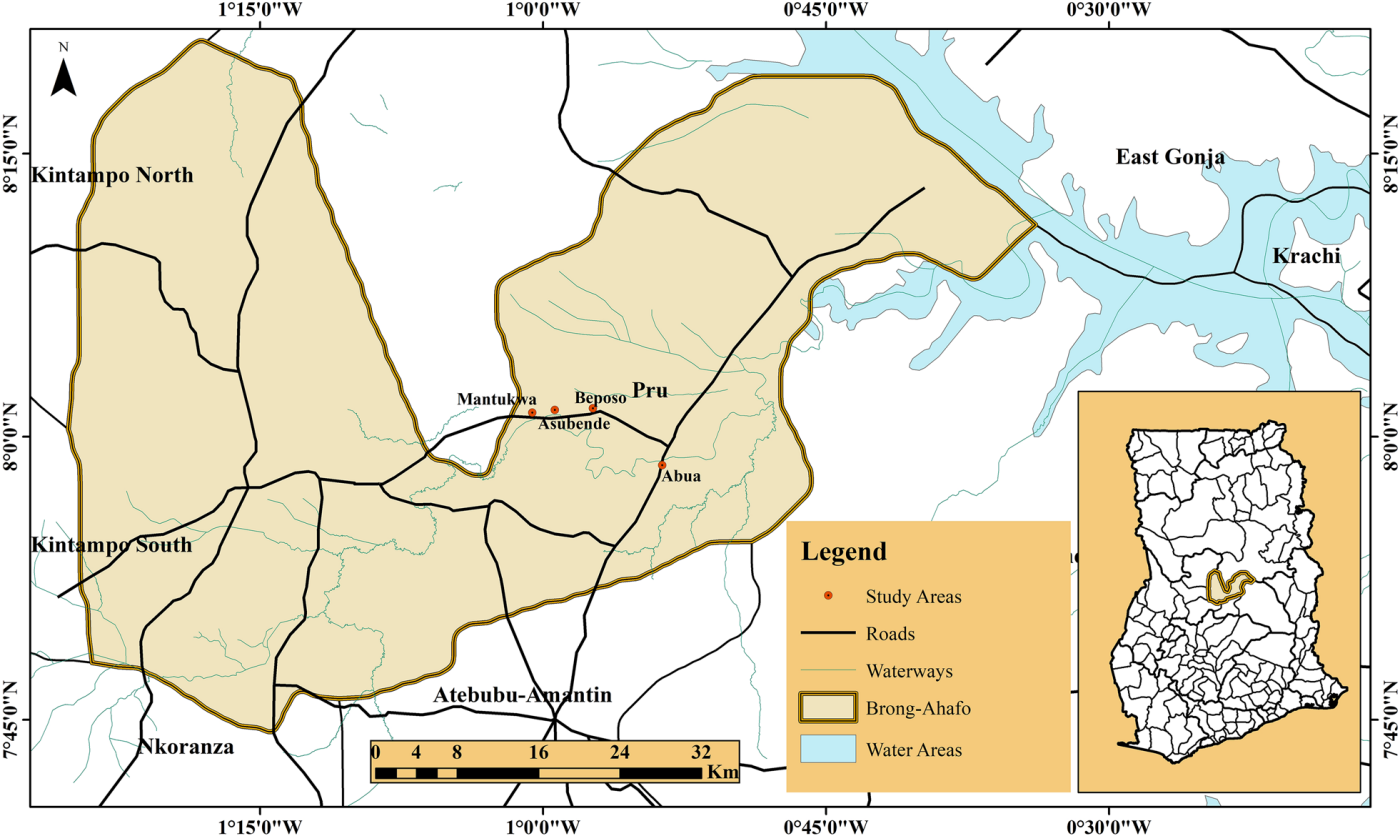
Map of the Pru district showing study communities as well as boundaries with other surrounding districts

The studies were conducted in four first-line onchocerciasis endemic communities located at least 5 km away from each other along the Pru River basin which is the major breeding site in the area. The names and Geographical Positioning System (GPS) location of the communities are: Abua-I (07°58′N, 000°53′W), Asubende (08°01′N, 00°58′W), Beposo (08°01′N, 000°57′W) and Mantukwa (08°01′N, 001°00′W), all located in the Pru district of the Brong-Ahafo region, Ghana. The Pru district lies between latitudes 07°50′N and 08°22′N and longitudes 00°30′W and 01°26′W, with a land area of 2,195 km^2^. It shares boundaries with Salaga district to the North, Atebubu-Amantin and Nkoranza districts to the South, Sene district to the East and Kintampo South and Kintampo North districts to the West (Fig. 1). The district lies within the Guinea-savanna bioclimatic zones of West Africa with two major seasonal variations in weather: the rainy and dry seasons.

### Collection of man-biting adult black flies

Six sets of entomological surveys were conducted, three in the rainy season and three in the dry season in each of the four communities. The rainy season (May-October) data were collected in the months of July, August and October (2012) while data from the months of January, February and March represented the dry season (November 2012-April 2013). For each month, flies were collected for four consecutive days in each community at designated black fly catching points located along the banks of the Pru River using human landing method [39]. Two consented collectors from each community were trained for vector collection. They worked alternately in each community between 06:00 and 18:00 GMT. Each seated vector collector wore protective clothing which allowed only their legs to be exposed. To minimize the effects of skin colour among the collectors, dark-skined persons were selected from among members of each local endemic community. Care was also taken to capture the black flies before they could obtain a blood meal. Female adult flies were caught individually into plastic catching tubes. Flies collected on hourly basis were labeled to indicate the hour, date, and place of capture. Flies were kept alive by wrapping the collection tubes containing the flies with moist cotton wool and placed in a cool environment, and later transported in ice chest to a makeshift field laboratory for further analysis. 

### Larval collection

Larval prospection for cytological studies was conducted in two communities: Asubende and Mantukwa because rapids and exposed rocky surfaces that supported fly breeding as at the time of these studies were found only in these communities. Prospection involved careful inspection of substrates such as overhanging and submerged plants, rocks, and twigs in the breeding sites for samples of preimaginal (aquatic) black fly stages. Larvae were preserved in freshly prepared Carnoy’s fixative (one part glacial acetic acid in three parts absolute ethanol) contained in universal bottles and transported in ice to a makeshift field laboratory and stored at 4ºC for future karyotyping. This aspect of our study was to serve as a confirmatory study for the finding from our preliminary morphological assessments to ascertain the species mix in the study area. 

### Morphological identification and dissection of adult black flies

Adult flies were distinguished morphologically into savanna and forest species using color variations of the antennae, scutella setae, arculus, wing tuft and the ninth abdominal tergite setae in line with previously published morphological taxonomic keys [40, 41]. Savanna flies were characterized by pale colors of the first three antennal segments, scutella satae, the wing tufts and the 9th abdominal tergite satae whereas their forest counterparts show darker colors of the aforementioned features. All flies collected were anaesthetized individually with chloroform vapor to immobilize them before they were dissected. Each fly was placed dorso-ventrally on a microscope slide in a drop of physiological saline and dissected from the abdomen to assess their parity status. Parous flies were identified exclusively by the presence of ovarian relics; retained eggs, pale coloured Malphigian tubules, absence of fat bodies and elasticity of the ovariole follicles were also used to infer parity [42, 43]. A parous fly, one that had taken at least one blood meal and had completed at least one gonotrophic cycle, resulted in the presence of follicular relics below the maturing oocyte [42, 43], whereas nulliparous flies had not yet taken a blood meal and had not completed any gonotropic cycle as indicated by the tightly coiled ovarian tracheal systems, absence of follicular relics. The head, thorax and abdomen of all parous flies were then dissected separately and the number of all third stage infective L_3_ and other developmental stages (L_2_ and L_1_) were recorded. Data obtained from adult black flies dissection were used to estimate entomological parameters such as parity and transmission potentials.

### Preparation and staining of *Simulium* larvae

Polytene chromosome slides were prepared using the methods previously described by Dunbar and Vajime [44] with minor modifications. The larvae were stained in lacto-acetic orcein for a period between 3 and 12 hours. In cases where the larvae had been stored for more than 3 months, they were placed in 1M HCl for 1 minute and washed 3 times in distilled water before staining. Full karyotyping and cytospecies identifications were based on the criteria of Boakye [11], Boakye et al. [12] and Post et al. [45]. The chromosome complements were read under a compound microscope to visualize the banding patterns, inversion breakpoints and new inversions used for the identification of each larva following previously described criteria [11, 12, 45].

### Estimation of entomological indices

The monthly biting rates (MBR) were measured as the theoretical number of *Simulium* bites received by a person who remained at a catching site during the 12 hours of the daylight for one complete month in a community. Similarly, the monthly transmission potential (MTP) was estimated as the total number of infective L_3_ larvae inoculated into one person in 1 month at a fly catching point during the 12 hours of the daylight period in a community [22, 46].

### Data analysis

The statistical differences in monthly relative abundances of members of the *S. damnosum* s.l. were estimated using the two-way analysis of variance (ANOVA) in a GENSTAT statistical software version 11 at the 5% level of significance. The difference in infection rate as well as the relationship between season and fly infectivity rates were also estimated using the Chi-square (*χ*^2^) test.

## Results

### Relative abundance of adult female black flies

A total of 9,983 female adult black flies were caught in the four communities during the entire study period, 6,569 (65.8% of total) were collected during the rainy season months of July, August and October while 3,414 (34.2% of total) were caught during the dry season months (January–March) (Table 1). The highest (2,870 flies) and lowest (876 flies) were captured in the months of July and January, respectively. Among the four study communities, the highest number of flies collected during the rainy season was recorded at Beposo, and the highest number of flies caught during the dry season was recorded at Asubende; however, when total collection was considered, more flies were recorded at Asubende than at Beposo. In contrast, the lowest number of flies captured in both seasons was recorded at Abua-I. A comparison of the relative abundances of black flies caught during the entire study period showed that a significantly higher number of flies were captured during the rainy season compared to the dry season (*F* = 0.021, *df* = 1, *P* < 0.05) (Table 1).

**Table 1:** Monthly relative fly abundance for the four study communities throughout the study period

Catching sites	Number of flies caught according to month						Total n flies captured according to community	Percentage flies caught/site
	July	August	October	January	February	March		
Abua I	170	35	42	19	44	99	409	4.09
Asubende	799	802	666	425	612	628	3932	39.38
Beposo	1365	876	464	250	348	252	3555	35.61
Mantukwa	526	414	410	182	288	267	2087	20.90
Total	2870	2127	1582	876	1292	1246	9983	100
Percentage monthly catches	28.7	21.3	15.8	8.8	12.9	12.5		

### Diurnal biting rate

**Fig. 2:**
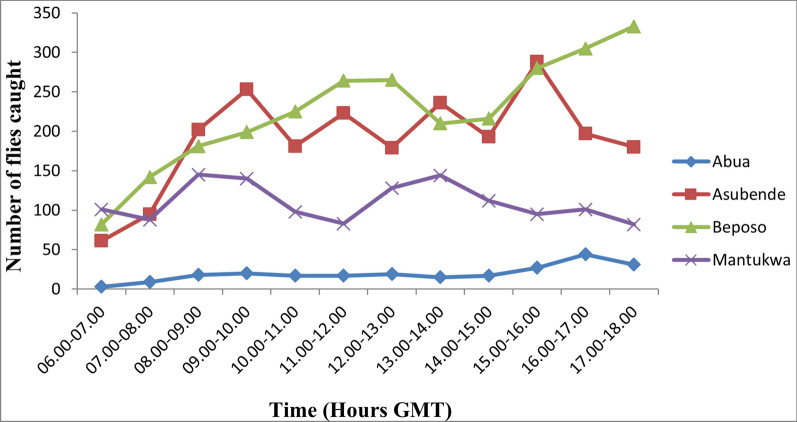
Diurnal biting rates of the *Simulium damnosum* complex in the four study communities during the wet season. GMT, Greenwich Mean Time

**Fig. 3:**
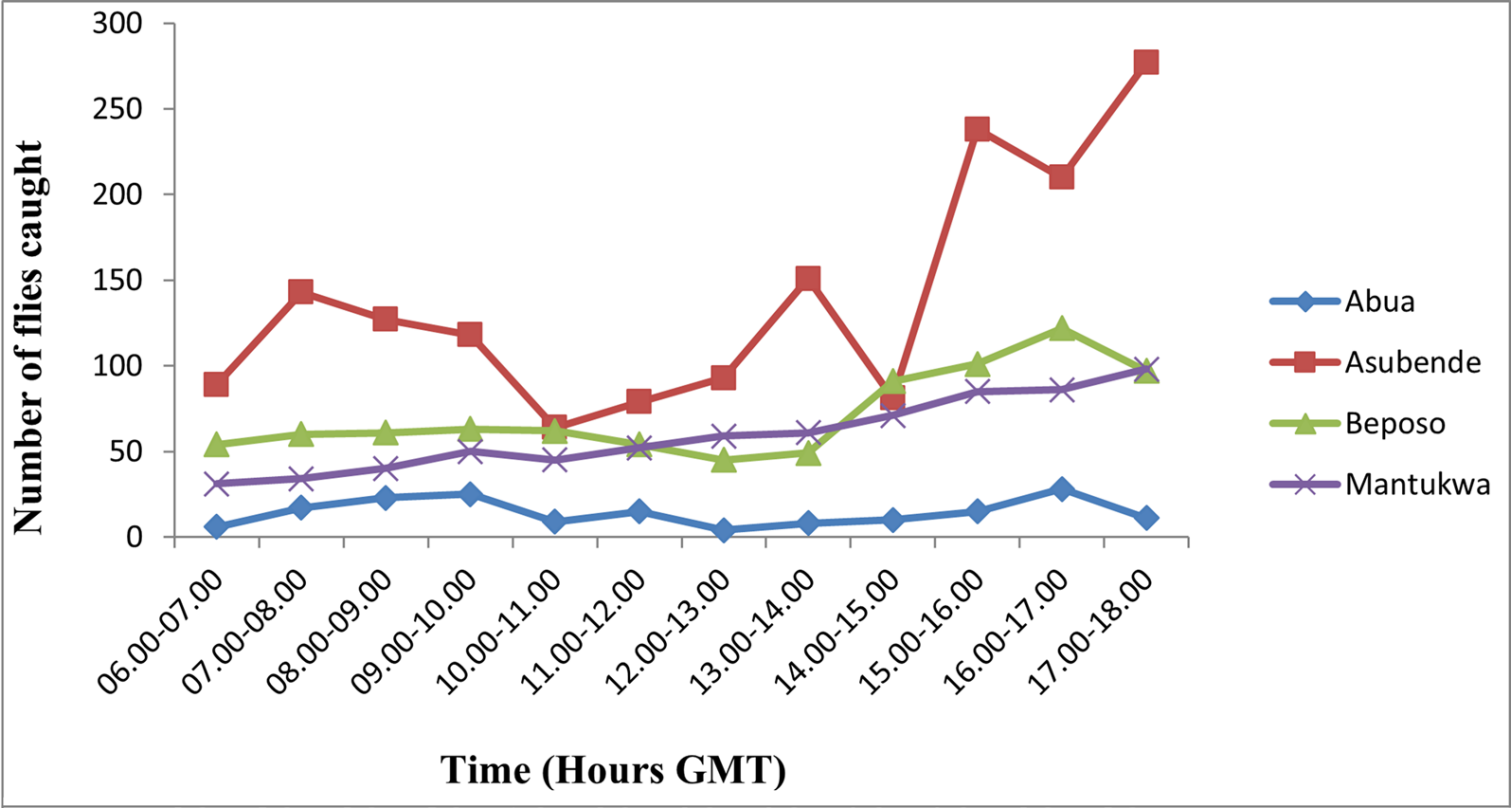
Diurnal biting pattern of *S. damnosum* complex in the four study community during the dry season. GMT, Greenwich Mean Time

Hourly variations in diurnal pattern of fly biting activities were observed across the four communities during the study period. In general, fly biting activities showed a characteristic bimodal pattern, with a morning peak occurring at different hours of the day, but mostly between 09:00 and 10:00 GMT in the mornings, and an evening peak between 16:00 and 17:00 GMT. Depending on the weather and other ecological conditions, a third peak was observed between 11:00 and 13:00 GMT or even multiple peaks of biting activities (see Fig. 2). In communities like Abua-1, Beposo and Mantukwa, the biting peaks were less distinct, while in the Asubende community multiple peaks were recorded, with distinct early morning peaks between 9:00 and 10:00 GMT and evening peak between 15:00 and 16:00 GMT (Figs. 2, 3).

**Fig. 4:**
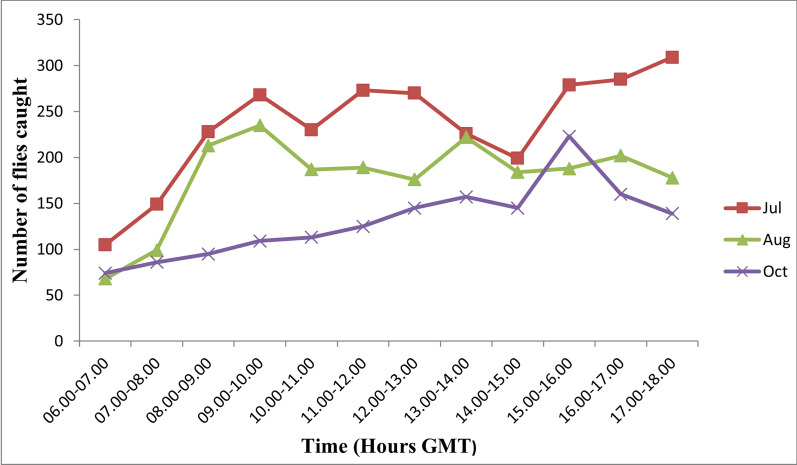
Diurnal biting pattern of *S. damnosum* complex for the three wet months in the study communities. GMT, Greenwich Mean Time

**Fig. 5:**
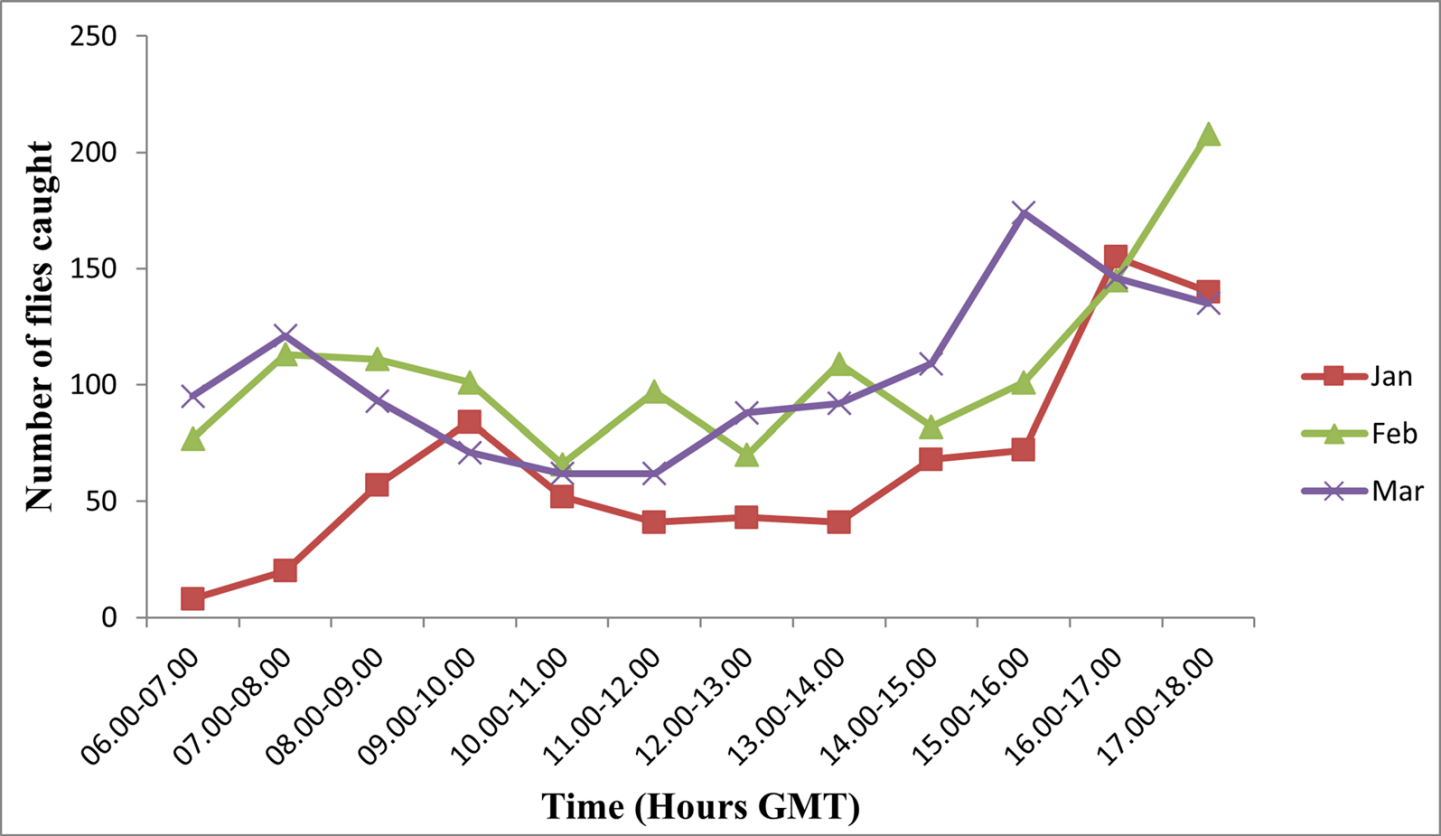
Diurnal biting pattern of *S. damnosum* complex for the three dry months in the study communities. GMT, Greenwich Mean Time

There were three peaks of biting activities in the month of July: between 09:00 and 10:00, between 11:00 and 12:00 and between 15:00 and 17:00 GMT. Two peaks of biting activity were recorded in August: between 09:00 and 10:00 and 13:00 and 14:00 GMT. Only one peak of biting activity was recorded in October: between 15:00 and 16:00 GMT. Fly activities in January peaked between 09:00 and 10:00 and between 16:00 and 17:00 GMT. A pronounced fluctuation in fly biting activities was recorded in February, with peak biting intensities occurring between 07:00 and 08:00, between 09:00 and 10:00, between 11:00 and 12:00 and between 13:00 and 14:00, and two distinct peaks occurring between 16:00 and 17:00 GMT. Fly biting activities in March also exhibited a bimodal pattern, with peaks between 07:00 and 08:00 and between 15:00 and 16:00 GMT. The minimum number of flies biting humans was recorded between 06:00 and 08:00 GMT while the maximum catches occurred between 16:00 and 18:00 GMT. The hourly and seasonal variations in fly numbers in the four study communities are shown in Figures 2, 4 and 5. The diurnal biting activities of black flies among the wet months as well as those between the dry months were not significantly different from each other (*F* = 0.058, *df* = 2, *P* > 0.05). However, the difference in diurnal biting activities between the dry months and the wet months was found to be significantly different (*F* = 0.04, *df* = 2, *P* < 0.05).

### Monthly biting rates

**Fig. 6:**
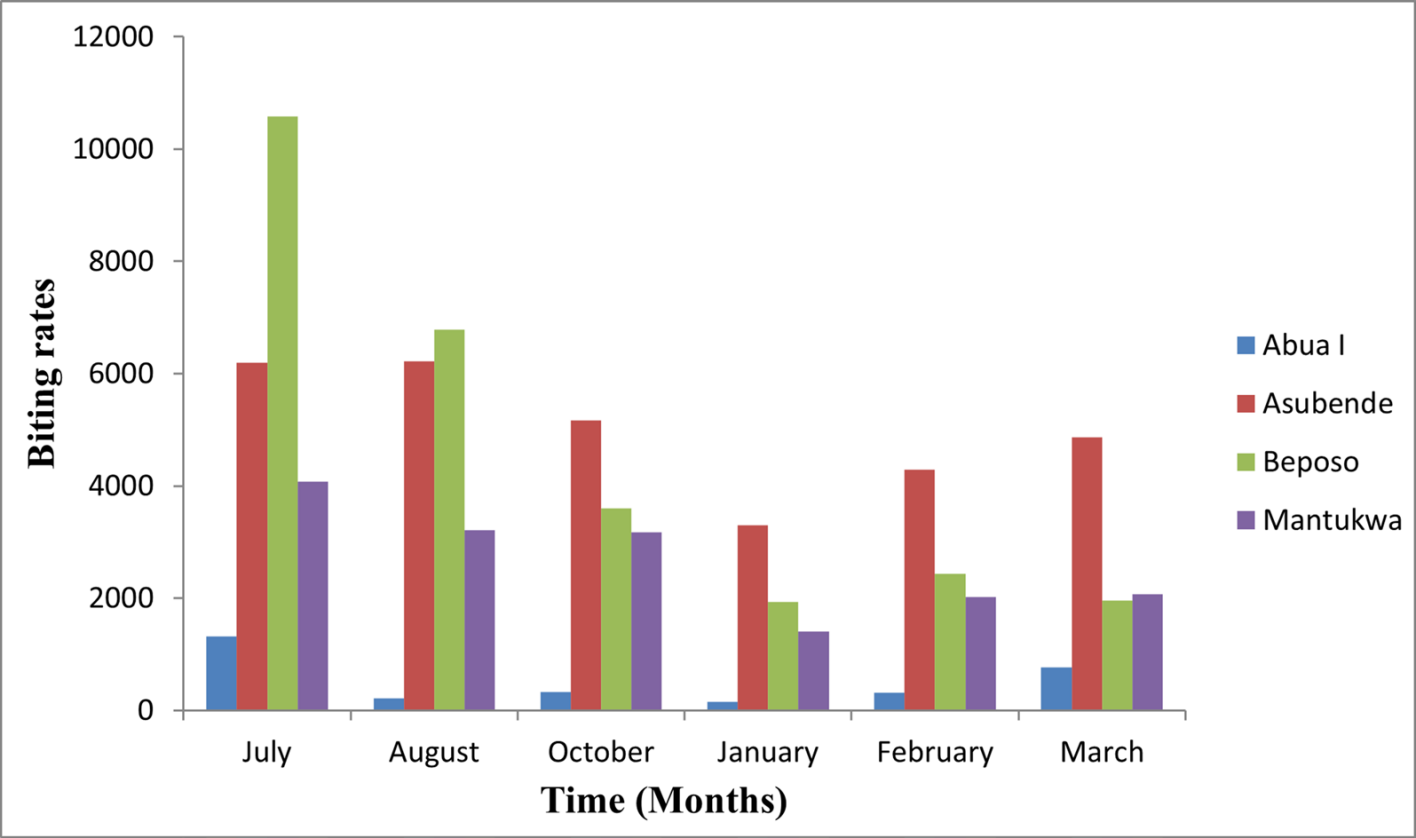
Summary of monthly biting rates in the four study communities

The MBR of black flies in each community were estimated for each month during the study period (Fig. 6). The highest MBR of 10,578.8 bites/person/month was recorded during the rainy month of July in Beposo, and the lowest MBR of 147.3 bites/person/month was recorded during the dry month of January at Abua-I. A comparison of MBR among the communities showed that Asubende, Beposo and Mantukwa had significantly higher MBR than Abua-1 and that Asubende and Beposo had significantly higher MBR than Mantukwa (*F* = 0.01, *df* = 3, *P* < 0.05); however, there was no significant statistical difference in MBR between Asubende and Beposo (*F* = 0.063, *df* = 1, *P* > 0.05).

A comparison of MBR in the rainy and dry seasons showed that MBR were significantly higher during the rainy months than during the dry months (*F* = 0.023, *df* = 2, *P* < 0.05). Among the rainy months, the MBR was significantly higher in July than in October, but not August. However, there was no significant difference in MBR among the dry months (*F* = 0.053, *df* = 2, *P* > 0.05).

### Parity rates

**Fig. 7:**
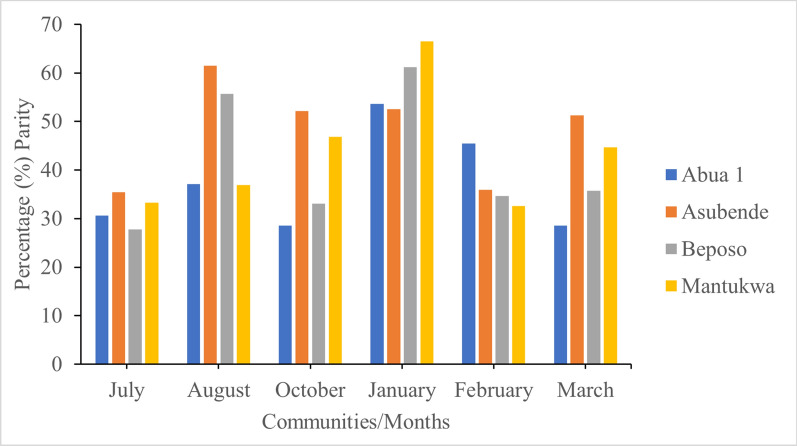
Percentage parity rates in the four study communities for the entire study period

All of the 9,983 flies collected from the four study communities for the purpose of this study were dissected for parity. A total of 4,197 (42.0%) flies were parous. All parous flies were further dissected to determine onchocerciasis infection rate. Among the parous flies dissected, 2,670 (63.6%) were collected during the rainy season and 1,527 (36.4%) were caught in the dry season. According to study site, the highest proportion (43.2%) of parous flies was collected in the Asubende community, and the lowest proportion (3.3%) was collected in the Abua-1 community; 33.0% and 20.4% of the parous flies were collected in the Beposo and Mantukwa communities, respectively. A seasonal consideration of parity rate indicated that the proportion of parous flies collected was highest in the dry season, especially in January when > 50% of flies dissected in all communities were parous. In the wet month of August however, a > 50% parity rate was recorded only in Asubende and Beposo, with the other communities (Mantukwa and Abua-1) having a < 50% parity rate in the same month. Analysis of the samples collected in October and March showed that only Asubende recorded a > 50% parity rate. During the months of July and February, all study communities reported parity rates of < 50% (Fig. 7).

### Monthly transmission potential

Of a total of 2670 (63.6%) parous flies collected in the rainy season that were dissected, 45 (1.7%) were infected with various developmental stages of *Onchocerca* larvae which were morphologically indistinguishable from *O. volvulus*, resulting in an infection rate of 1.7%. Of 45 infected flies, 27 harbored infective larvae (L_3_) in the heads (Table 2).

**Table 2:** Summary of transmission indices of *Simulium damnosum* in the four collection sites

Characteristics	Abua I	Asubende	Beposo	Mantukwa
Person’ days worked	24	24	24	24
Total* n* flies caught	409	3932	3555	2087
Average daily catch per person,* n*	17	164	148	87
*Number (%) of flies dissected*	409 (100)	3932 (100)	3555 (100)	2087 (100)
Number (%) of parous flies	140 (35.0)	1814 (46.1)	1385 (39.0)	858 (41.1)
Number (%) of nulliparous flies	269 (65.0)	2118 (53.9)	2170 (61.0)	1229 (59.9)
*Total number of flies infected (%)*	1 (0.2)	21 (0.5)	15 (0.4)	13 (0.6)
Number of flies (%) with L_1_ and L_2_	1 (0.2)	5 (0.1)	7 (0.2)	2 (0.1)
Number of flies (%) with L_3_ in theHead	0 (0)	16 (0.4)	8 (0.2)	11 (0.5)

*L*_*1*_,* L*_*2*_,* L*_*3*_ First-, second- and third-stage larvae, respectively

**Fig. 8:**
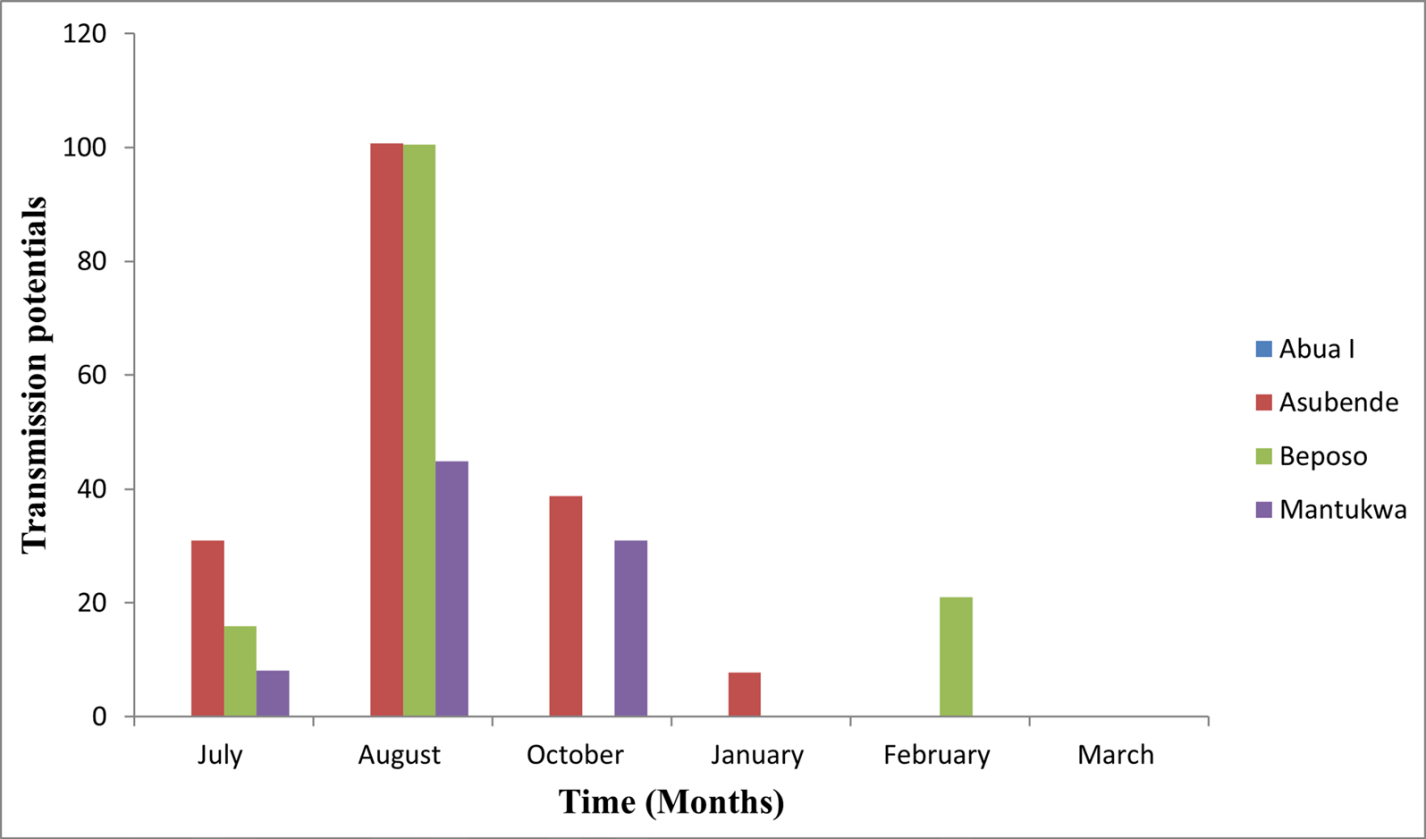
Summary of monthly transmission potentials in the four collection sites

The overall *Onchocerca* infection rates during the months of the dry season were very low as compared with the wet season. Of the 1,527 flies dissected in the dry season, only five (0.3% of flies caught) were infected and only two flies had infective L_3_ in the head. Our results showed different levels of *O*. *volvulus* transmission in the study communities, with the highest transmission potential of 101 infective bites/person/month recorded in Asubende and no. transmission of *O*. *volvulus* recorded in the Abua-1 throughout the study period. Onchocerciasis transmission was generally low during the dry season, occurring only in Asubende and Beposo in the months of January and February, respectively, with transmission potentials of 7.8 and 21.0 infective bites/person/month (Fig. 8).

### Morphological identification of adult black flies

A preliminary investigation was conducted using a random sample of 2,032 flies collected from the four study communities to ascertain whether members of the forest and savanna species of the *S*. *damnosum* complex coexist in the study communities. The morphological identificationof forest and savanna species was based on a combination of color variations. The color of the fore coxae was not included in the analysis since some populations of *S. squamosum* share this feature in common with members of both *S. damnosum* s.s. and *S. sirbanum*, especially in the eastern parts of the former Onchocerciasis control program (OCP) area in West Africa. However, the color of the first three antennal segments, the color of the wing tuft and the color of the ninth abdominal tergite setae were used to separate forest species from their savanna counterparts. In all, 2.028 flies were scored with pale wing tuft, pale antennae and pale ninth abdominal tergite setae, which are characteristics of the savanna species of the *S*. *damnosum* complex, and were therefore designated as savanna flies. In comparison, only four flies were characterized by the dark colors of all these aforementioned anatomical parts and were thus identified as forest flies.

### Cytotaxonomic identification

Given the determination of the presence of forest cytospecies in our study area, we employed cytological tools to further confirm whether these forest flies were inhabiting this savanna area or were just on a mere host-seeking expedition in the study area. The polytene chromosomes of a total of 656 *Simulium* larvae collected from breeding sites located at Asubende and Mantukwa were stained and karyotyped for cytological studies. However, only 114 larvae produced readable chromosomes, among which 73 were sampled in the rainy season (43 from Asubende and 30 from Mantukwa). Twenty-nine of the samples collected from Asubende were identified as *S. damnosum* s.s. based on the diagnostic inversion IIL-C/C or IIL-C/C.8 and the rest were identified by the inversions IIL-C.8/C.8, IIL-C.8.3/C.8.3 or IIL-C.8/C.8.3 as *S. sirbanum*. In the Mantukwa area, 19 samples were identified as *S. damnosum* s.s. while 11 were identified as *S. sirbanum*. Thus, all larvae karyotyped in the wet season were savanna flies and there were no records of forest species in the wet season. During the dry season, however, there was no fly breeding at Mantukwa presumably because the flow rate of the rapids at the breeding sites was insufficient to support fly breeding. Therefore, all 41 larval samples karyotyped were collected from the Asubende breeding site, of which 24 were identified as *S. damnosum* s.s. and 16 as *S. sirbanum*, all based on the aforementioned diagnostic inversions. A single specimen was identified from Asubende as *S. sanctipauli* sensu lato (*S. sanctipauli* s.l.) in the dry season.

Some of the inversions recorded in this study include IS-3, IS-2, IS-18, IS-22, IIL-C/C, IIL-C.8/C.8, IIL-C/C.8, IIL-4, IIIL-2, among others. Members of the *S. damnosum* s.s. were identified by the diagnostic inversion IIL-C/C or IIL-C/C.8; *S. sirbanum* was identified by the inversions IIL-C.8/C.8, IIL-C.8.3/C.8.3 or IIL-C.8/C.8.3. The single larva of *S. sanctipauli* s.l. found was diagnosed by the presence of the inversion IIL-4 (Additional file 1: Figures S1–S10).

## Discussion

The dual focus of this study is the transmission status of *O. volvulus* and *Simulium* species composition in some selected communities in Ghana. Such data is crucial in view of the onchocerciasis endemicity of the study areas specifically, and Ghana in general [38]. Moreso, all our study communities had received annual MDA with ivermectin for about two decades and are now under the biannual IVM treatment strategy with an average of about 80% treatment coverage, making it necessary to assess the impacts of these efforts on onchocerciasis transmission in these areas.

Our results showed seasonal variation in the relative abundance of flies caught. Fly populations in three of the study communities were significantly higher during the rainy season than the dry season. However, fly populations in Abua-1 were stable in both seasons, possibly because fly numbers remained small in this site and did not show much variation. The distribution and population density of black flies in the study areas varied considerably across seasons, possibly influenced by an increase in the number of breeding sites in the wet season as river volumes increased and submerged more trailing vegetations, logs, rocks and any other substrates that may later serve as points of attachment for immature black flies. In Abua-1 where the fly population appeared to be stable in both seasons, a cursory observation of the river system within the Abua-1 catchment area showed neither sufficient rapids nor rocky surfaces, both of which are essential to support breeding, possibly explaining, at least in part, our observations in the area. Our reports of seasonal variations in fly biting rates in relation to water level and ecological conditions are in agreement with the reports of Le Berre [47]. The flies collected from Abua-1 might have been foraging for blood meals but not necessarily breeding there.

Generally, in Asubende, Beposo and Mantukwa, the flies exhibited bimodal diurnal biting patterns peaking at different times of the day but mostly in the morning between 9:00 and 10:00 GMT and in the evening between 16:00 and 17:00 GMT. These periods of intense biting correspond to the periods when most individuals in the communities are actively engaged in their farming and fishing activities and are maximally exposed to the bites of black flies. This has significant epidemiological implications for disease transmission. This situation was worse in all the communities during the rainy season when fly populations were high, as also reported by other studies [48, 49], showing that more individuals were likely to acquire infections during the rainy season in most rural endemic communities.

The proportion of parous black flies peaked during the rainy season compared to the dry season, suggesting that parasite transmission may be more pronounced during periods of increased rainfall. The increased parous rate in the rainy season increases the risk of onchocerciasis transmission in the areas as more flies are likely to live long enough to transmit the disease. This finding is consistent with that of Boatin et al. [50] in the southern rainforest onchocerciasis focus of Nigeria, but in contrast to those of the WHO [51] in southwestern Nigeria and [24] in parts of the Brong-Ahafo region of Ghana.

Active parasite transmission occurred in the Asubende, Mantukwa and Beposo communities where we reported several flies harboring the infective L_3_ in their heads. The assessment of fly infectivity rates (one of the key determinants of interruption of *O. volvulus* transmission) showed that the highest fly infectivity rate was recorded in Asubende, followed by Mantukwa and Beposo. No infective flies were recorded from Abua-1 throughout the study. The significant infectivity rates of onchocerciasis vectors recorded in three of the communities provides strong evidence of on-going transmission. The WHO stipulated that a vector infectivity rate of < 1 L_3_ per 1000 parous flies is required to interrupt transmission of *O*. *volvulus* [23, 52]. Therefore, the vector infectivity rates in all three communities (Asubende, Beposo and Mantukwa) greatly exceeded the WHO’s recommended threshold for transmission interruption by more than fivefolds. Also, there have been reports of significantly persistent microfilaridemia as well as reports on suboptimal responses to IVM treatment in some of these endemic foci [53–55]. These findings have been further reinforced by the more recent findings of Frempong et al. [56].

Our results, when compared with those of other published studies, seem to suggest the interruption of *O*. *volvulus* transmission at Abua-1, but we urge extreme caution when interpreting this information because the data on fly numbers reported here were insufficient to draw statistically meaningful conclusions on Abua-1; molecular confirmatory studies are required to draw reliable conclusions. It must be recognized that even our reported biting rates for Abua-1 were below the threshold biting rates at which infection transmission may not be detected [57]. Earlier surveys conducted in Asubende in 2005 recorded infectivity rates of 1.82 infective larvae/1000 parous flies [38]. However, in the present study we found a fourfolds increase in fly infectivity rate—at 8.9 infective flies/1000 parous flies in the same community and this trend is worrisome given the continual biannual distribution of IVM in the area. The gradual build-up of fly infection rates in Asubende and the continued transmission of onchocerciasis in three out of the four study communities despite continuous MDA with ivermectin may be attributed to a number of factors, including a gradual increase in the number of human reservoirs of infection as a result of consistent noncompliance of individuals to IVM treatment; possible presence of other *Onchocerca* species like *O. ochengi* which are capable of infecting and developing in the *S*. *damnosum* s.l., leading to overestimation of *O*. *volvulus* transmission potentials; earlier than expected skin repopulation by *O. volvulus* from individuals responding sub-optimally to IVM treatment; and immigration of individual human hosts with high microfilaridemia from untreated hyper-endemic communities into already treated areas.

In line with the WHO’s Roadmaps for Neglected Tropical Diseases (NTDs), Ghana has currently changed its treatment strategy from annual to biannual treatment to meet the current goal of elimination of infection in selected African countries [58]. This involves mass distribution of IVM at intervals of 6 months in selected hyper-endemic and meso-endemic communities aimed at effectively reducing the prevalence and intensity of infection to levels where the infection is not self-sustaining, ultimately leading to the interruption and elimination of *O. volvulus* infection. To attain these targets, there is the need to improve and strengthen existing logistic support systems for community volunteers, who are the lifeline for the community directed treatment with ivermectin (CDTi). This will ensure that community treatments are carried out at stipulated time periods to maximize treatment compliance and improve treatment coverage [59]. Since maximum parasite transmission were reported in the months of July and August, it will be extremely essential to schedule the timings of IVM treatments to take advantage of the impact of IVM on transmission. Ivermectin MDA should be conducted just before these periods of peak parasite transmission to ensure maximum effect on parasite transmission intensity. Alternative strategies, such as testing for onchocerciasis and treating those still infected with anti-*Wolbachia* drugs (such as doxycycline), and vector control strategies, such as the ‘Slash and Clear’ technique targeting larvae in the breeding site, could be employed to facilitate onchocerciasis elimination.

Accurate vector identification is an essential tool in the planning, execution, management and evaluation of vector control programs. Previous information on the species diversity of members of the *S*. *damnosum* complex involved in the transmission of *O. volvulus* in the Asubende catchment area is available [24], but the data in this study are not based on cytotaxonomic analysis, which remains the most reliable means of species identification in the Simuliidae [11]. Moreover, following our initial report of forest species in the study area, further cytological descriptions of members of the *S. damnosum* complex breeding at Asubende and Mantukwa were conducted using the criteria provided for West Africa simuliid species [11, 12]. Similar guides have been published for other parts of Africa [28, 60]. The members of the *S. damnosum* complex sampled from the study areas conformed to the descriptions by Boakye [11], Boakye et al. [12] and Post et al. [45]. *Simulium. damnosum* s.s. and *S. sirbanum* are reported to have a widespread distribution across Ghana, especially in areas close to the study areas [14].

We report here that the savanna members (*S*. *damnosum* s.s. and *S*. *sirbanum*) of the *S. damnosum* subcomplex are the dominant species in the study areas. This result is in agreement with earlier findings [50, 52, 61], all of which showed that *S. sirbanum* and *S. damnosum* s.s. are the most widely distributed members of the *S. damnosum* complex in the former OCP area of West Africa. We also report for the first time, at least up to the time of this study, the occurrence of forest cytospecies in the savanna setting, which we confirmed by cytotaxonomic analysis. In West Africa (Ghana and Togo), there have been reports of savanna members of the *S*. *damnosum* s.l. invading forest areas that have undergone environmental degradation, but there had not been any such report of the forest species moving into savanna areas until our study. However, the circumstances surrounding the migration of forest flies into savanna areas have not been established [57] in the forest settings. It is not clear whether the presence of *S. sanctipauli* s.l. as recorded in Asubende has a long history or represents a recent invasion probably in search of suitable breeding sites. The confirmation of the presence of forest black fly species in Asubende by cytotaxonomy points to a likely establishment of this group of flies in the area. This is in agreement with reports from earlier studies of [35, 62] who also reported that *S. squamosum, S. yahense* and the Beffa form of *S. soubrense* coexisted with *S. damnosum* s.s. and *S. sirbanum* in some parts of Nigeria, although the forest species dominated in the forest bioclimes while the savanna species dominated in the savanna enclaves. It is possible that the forest fly (*S*. *sanctipauli*) had actually migrated to this area as there has never been any such record from this site, although Boakye et al. [14] did report the presence of *S. sanctipauli* from relatively nearby rivers. Such findings could be of significant epidemiology importance to onchocerciasis elimination in Ghana [61].

## Conclusions

The results of this study confirm the active transmission of onchocerciasis in the study communities, with the exception of the Abua-1 community. Our study also provides further information on the biting behaviors and onchocerciasis transmission indices of the *S. damnosum* complex among communities located along the Pru river basin, including Abua-1, Asubende, Beposo and Mantukwa, and shows a clearer trend in the current situation in the study communities, especially Asubende. This information generated will help provide the basis for continuous monitoring and evaluation of the impact of ongoing control programs on disease transmission in these areas. MTPs in all but one of the communities were greater than the WHO tolerable limits for interruption of *O*. *volvulus* transmission in hyperendemic areas. Since there has been a new paradigm shift targeted at eliminating *O*. *volvulus* infection from among selected African countries, including Ghana, effective evaluation of the impact of IVM MDA and monitoring for persistent infection transmission should be sustained to ensure that transmission levels are brought below transmission breakpoints to facilitate the achievement of the goals of onchocerciasis elimination. Further, our data have confirmed the species *S*. *damnosum* *complex* inhabiting the study area as savanna (*S. damnosum s.s.* and *S. sirbanum*) species as the principal vectors of onchocerciasis in the Pru river catchment area although there is the occasional influx of forest cytotypes alien to this area.

## Supplementary Information

**Additional file 1**:** Figure S1.** Chromosome II showing IIS-st/st, IIL-C.8.3/C.8.3 and IS/st inversions typical of *S. sirbanum*. C = centromere, B = balbiani ring, db = double bubble.** Figure S2.** Chromosome II showing IIL-C.8/C.8 homozygous inversion typical of *S. sirbanum*. C = centromere, B = balbiani ring, db = double bubble.** Figure S3.** Chromosome II showing IIL-C/C homozygous inversion typical of *S. damnosum* s.s. C = centromere, B = balbiani ring, db = double bubble.** Figure S4.** Chromosome I showing IS-3 heterozygous inversion and IS-2 homozygous inversions typical of *S. damnosum* s.s./*S. sirbanum*. C = centromere, NO = nuclear organizer.** Figure S5.** Short arm of chromosome I of *S. sirbanum*. C = centromere, NO = nuclear organizer.** Figure S6.** Chromosome III showing IIIL-2/2 and IIL-27 homozygous inversions typical of *S. damnosum* s.s./*S. sirbanum/S. dieguerense*. C = centromere.** Figure S7.** Chromosome III showing IIIL-2/2 & IIL-27 homozygous inversions typical of *S. damnosum* s.s.*/S. sirbanum/S. dieguerense*. C = centromere.** Figure S8.** Chromosome III showing IIIL-2/2.7 homozygous inversions typical of *S. damnosum* s.s.*/S. sirbanum* C = centromere.** Figure S9.** Chromosome II showing IIL-4 homozygous inversion typical of members of the *S. sanctipauli* s.l. C = centromere, B = balbiani ring, db = double bubble.** Figure S10.** Chromosome II showing IIL-C.8/C.8.64 and IS/st inversions typical of *S. sirbanum* C = centromere, B = balbiani ring, db = double bubble.

## Data Availability

All data related to this work have been included in the manuscript and associated files.

## Abbreviations

GMT: Greenwich Mean Time
GPS: Geographical Positioning System
HLC: Human landing collection
IVM: Ivermectin
L_1_: First-stage larvae of *Onchocerca volvulus*
L_2_: Second-stage larvae of *Onchocerca volvulus*
L_3_: Third-stage larvae of *Onchocerca volvulus*
MBR: Monthly biting rate
MDA: Mass drug administration
MTP: Monthly transmission potential
NTDs: Neglected tropical diseases
OCP: Onchocerciasis control program
