# Supplementary material for: Ongoing transmission of onchocerciasis in the Pru District of Ghana after two decades of mass drug administration with ivermectin and comparative identification of members of the Simulium damnosum complex using cytological and morphological techniques
Source: Parasit Vectors. 2024 Sep 18;17:394. doi: 10.1186/s13071-024-06333-2 (PMC11409746; doi:10.1186/s13071-024-06333-2)
Supplement: Supplementary file 1 — Additional file 1: Figure S1. Chromosome II showing IIS-st/st, IIL-C.8.3/C.8.3 and IS/st inversions typical of S. sirbanum. C = centromere, B = balbiani ring, db = double bubble. Figure S2. Chromosome II showing IIL-C.8/C.8 homozygous inversion typical of S. sirbanum. C = centromere, B = balbiani ring, db = double bubble. Figure S3. Chromosome II showing IIL-C/C homozygous inversion typical of S. damnosum s.s. C = centromere, B = balbiani ring, db = double bubble. Figure S4. Chromosome I showing IS-3 heterozygous inversion and IS-2 homozygous inversions typical of S. damnosum s.s./S. sirbanum. C = centromere, NO = nuclear organizer. Figure S5. Short arm of chromosome I of S. sirbanum. C = centromere, NO = nuclear organizer. Figure S6. Chromosome III showing IIIL-2/2 and IIL-27 homozygous inversions typical of S. damnosum s.s./S. sirbanum/S. dieguerense. C = centromere. Figure S7. Chromosome III showing IIIL-2/2 & IIL-27 homozygous inversions typical of S. damnosum s.s./S. sirbanum/S. dieguerense. C = centromere. Figure S8. Chromosome III showing IIIL-2/2.7 homozygous inversions typical of S. damnosum s.s./S. sirbanum C = centromere. Figure S9. Chromosome II showing IIL-4 homozygous inversion typical of members of the S. sanctipauli s.l. C = centromere, B = balbiani ring, db = double bubble. Figure S10. Chromosome II showing IIL-C.8/C.8.64 and IS/st inversions typical of S. sirbanum C = centromere, B = balbiani ring, db = double bubble. [file 13071_2024_6333_MOESM1_ESM.docx]

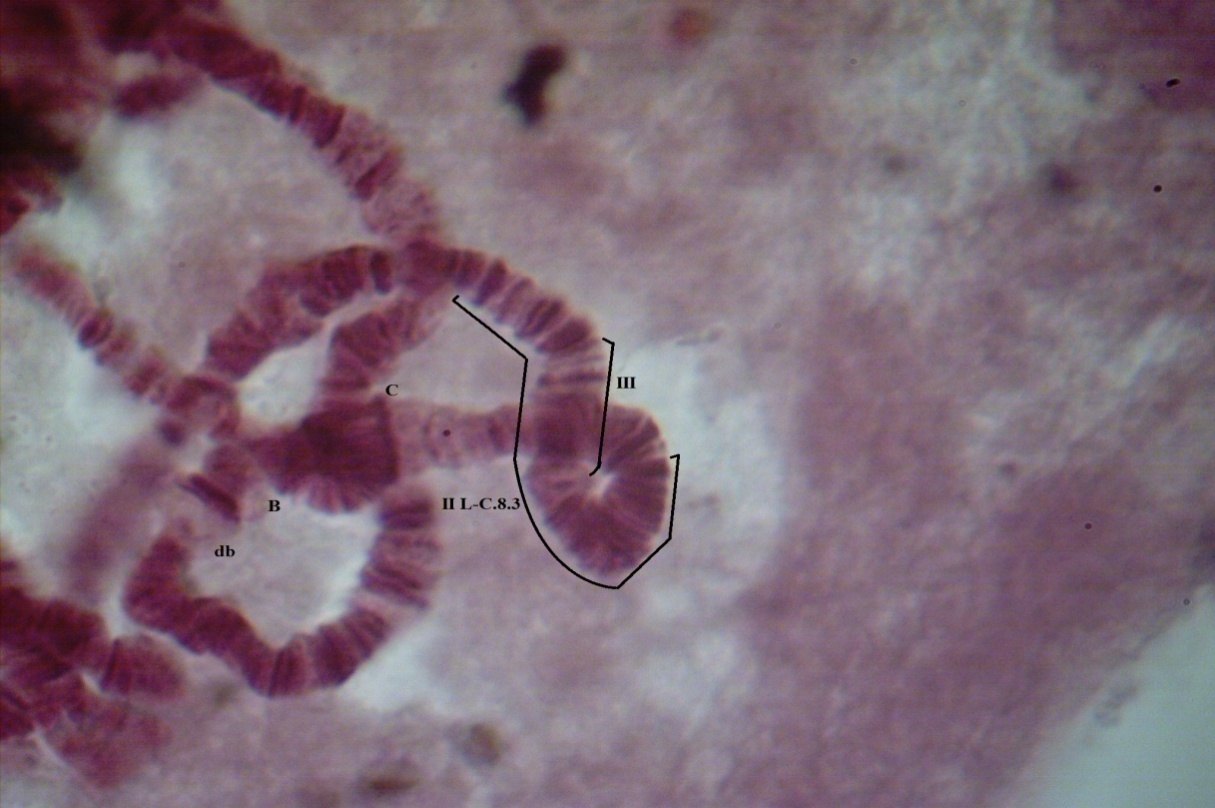
 **S 1**: Chromosome II showing IIS-st/st, IIL-C.8.3/C.8.3 and IS/st inversions typical of *S. sirbanum* C = centromere, B = balbiani ring, db = double bouble.


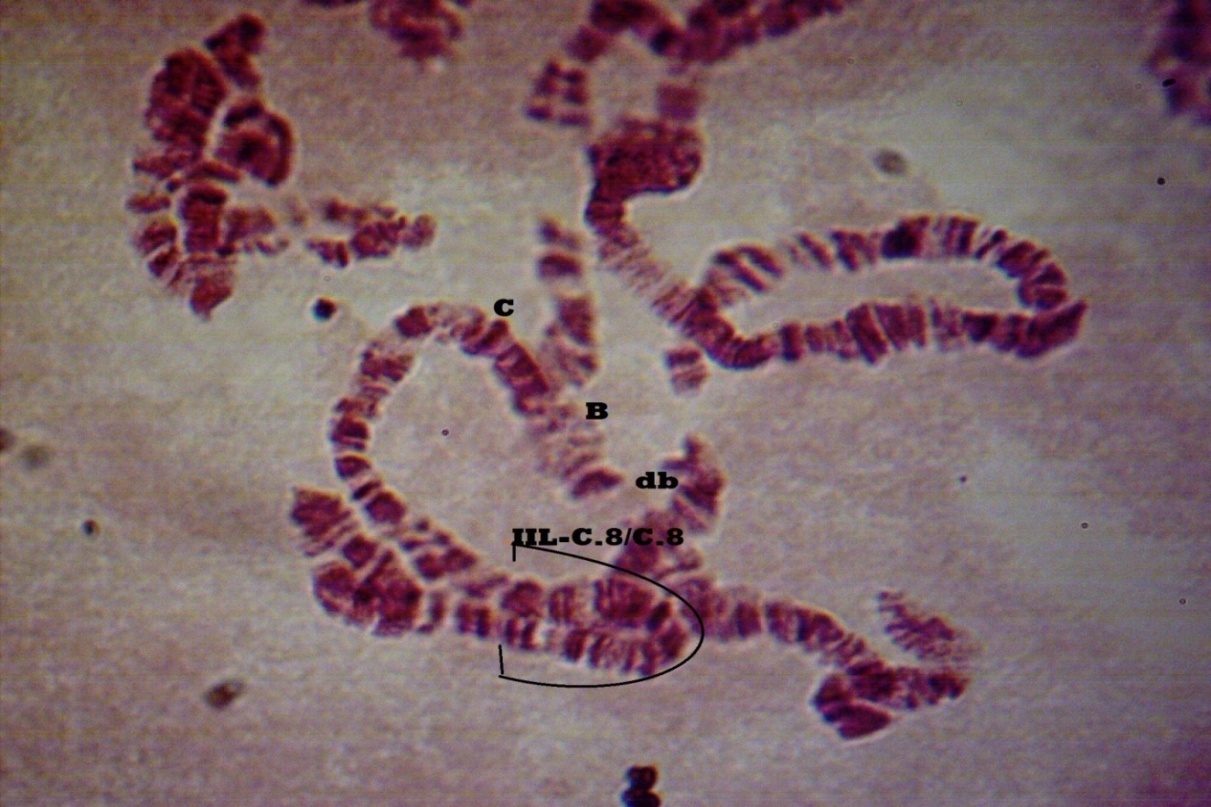
 **S 2:** Chromosome II showing IIL-C.8/C.8 homozygous inversion typical of *S. sirbanum*. C = centromere, B = balbiani ring, db = double bubble.


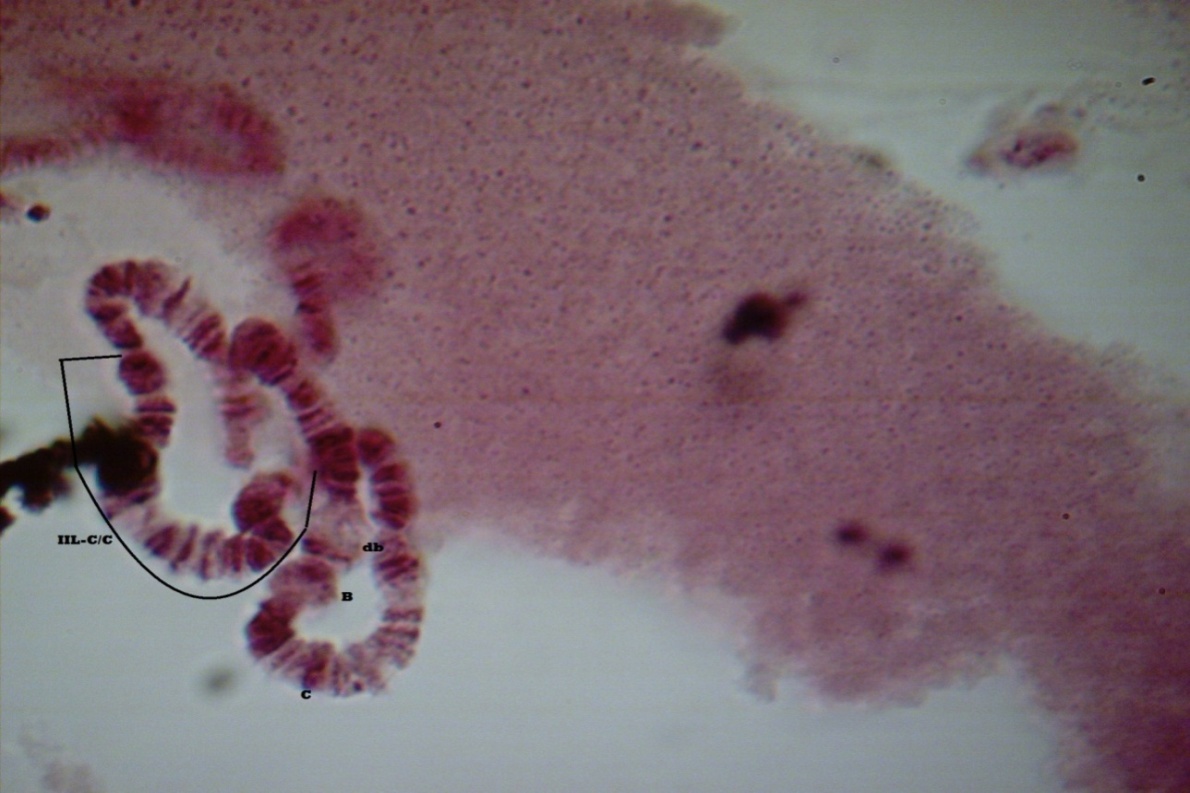
 **S 3:** Chromosome II showing IIL-C/C homozygous inversion typical of *S. damnosum* s. s. C = centromere, B = balbiani ring, db = double bubble.


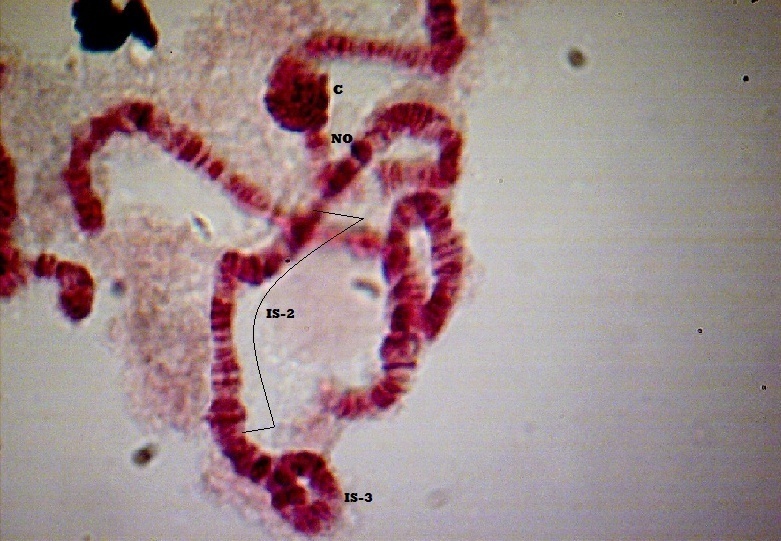
 **S 4:** Chromosome I showing IS-3 heterozygous inversion & IS-2 homozygous inversions typical of *S. damnosum* s.s/*S. sirbanum*. C = centromere, NO = nuclear organizer.


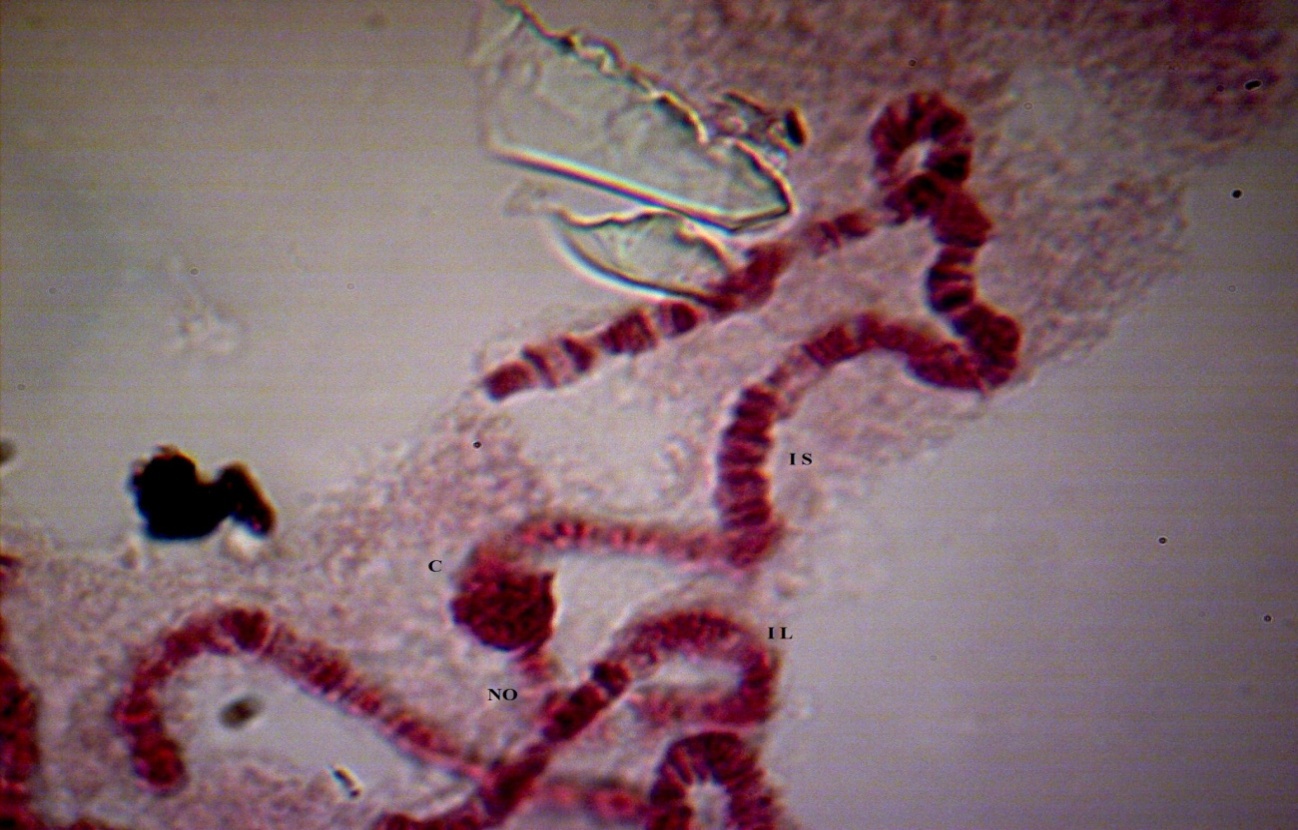
 **S 5:** Short arm of chromosome I of *S. sirbanum*. C = centromere, NO = nuclear organizer.


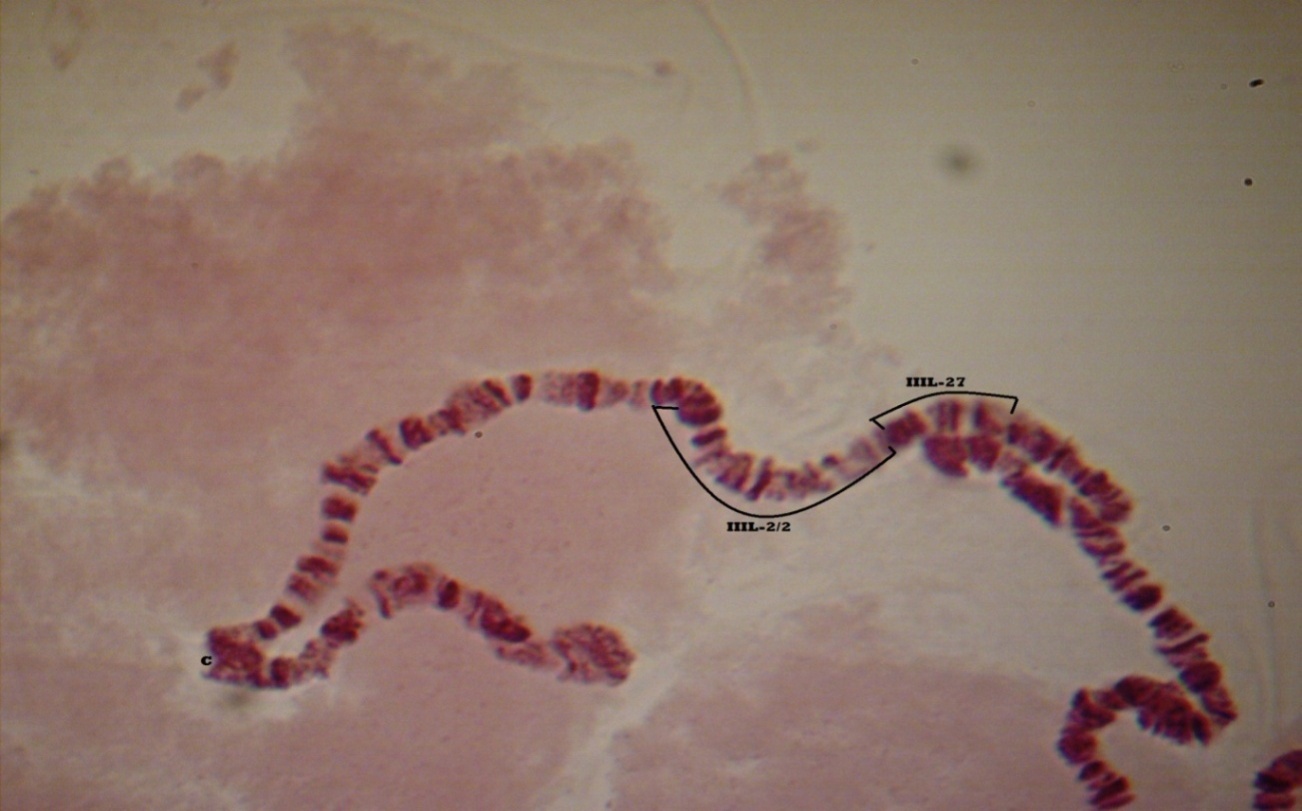
 **S 6:** Chromosome III showing IIIL-2/2 & IIL-27 homozygous inversions typical of *S. damnosum s.s./S. sirbanum/S. dieguerense*. C = centromere.


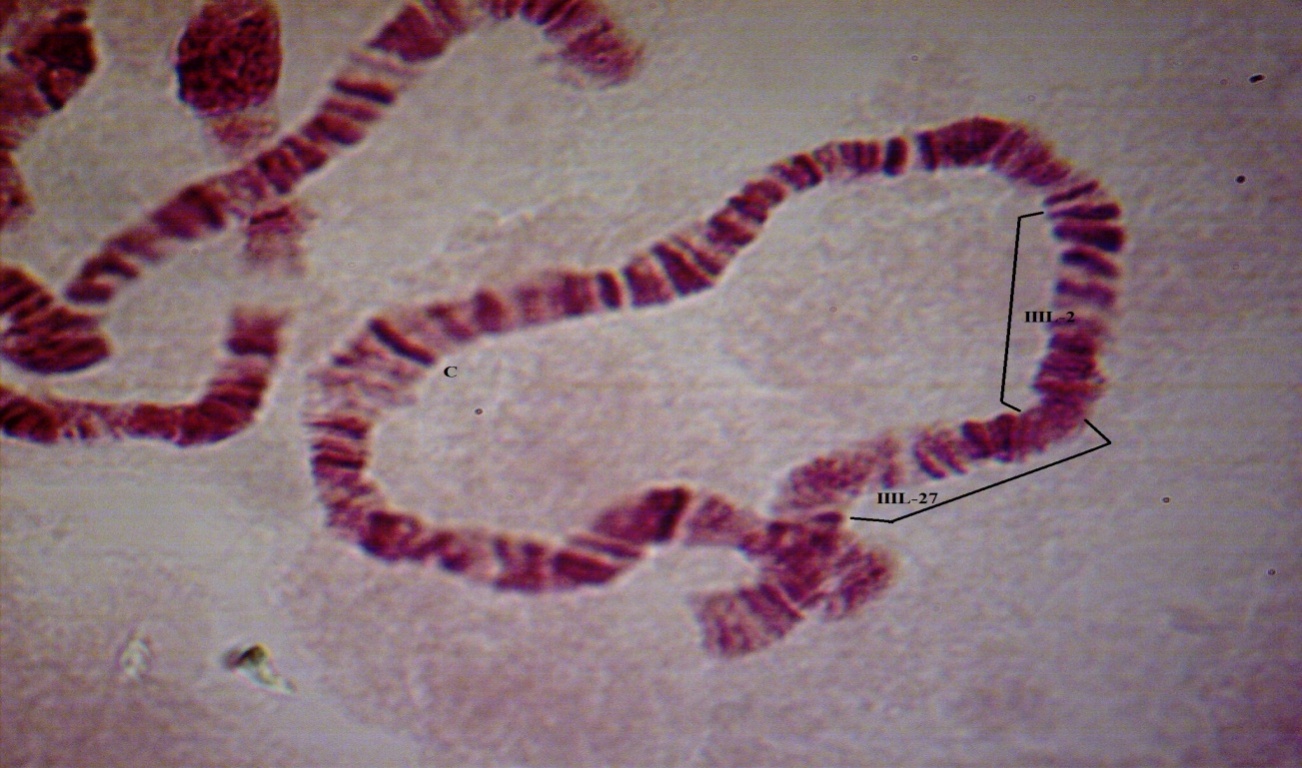
 **S 7:** Chromosome III showing IIIL-2/2 & IIL-27 homozygous inversions typical of *S. damnosum s.s./S. sirbanum/S. dieguerense*. C = centromere.


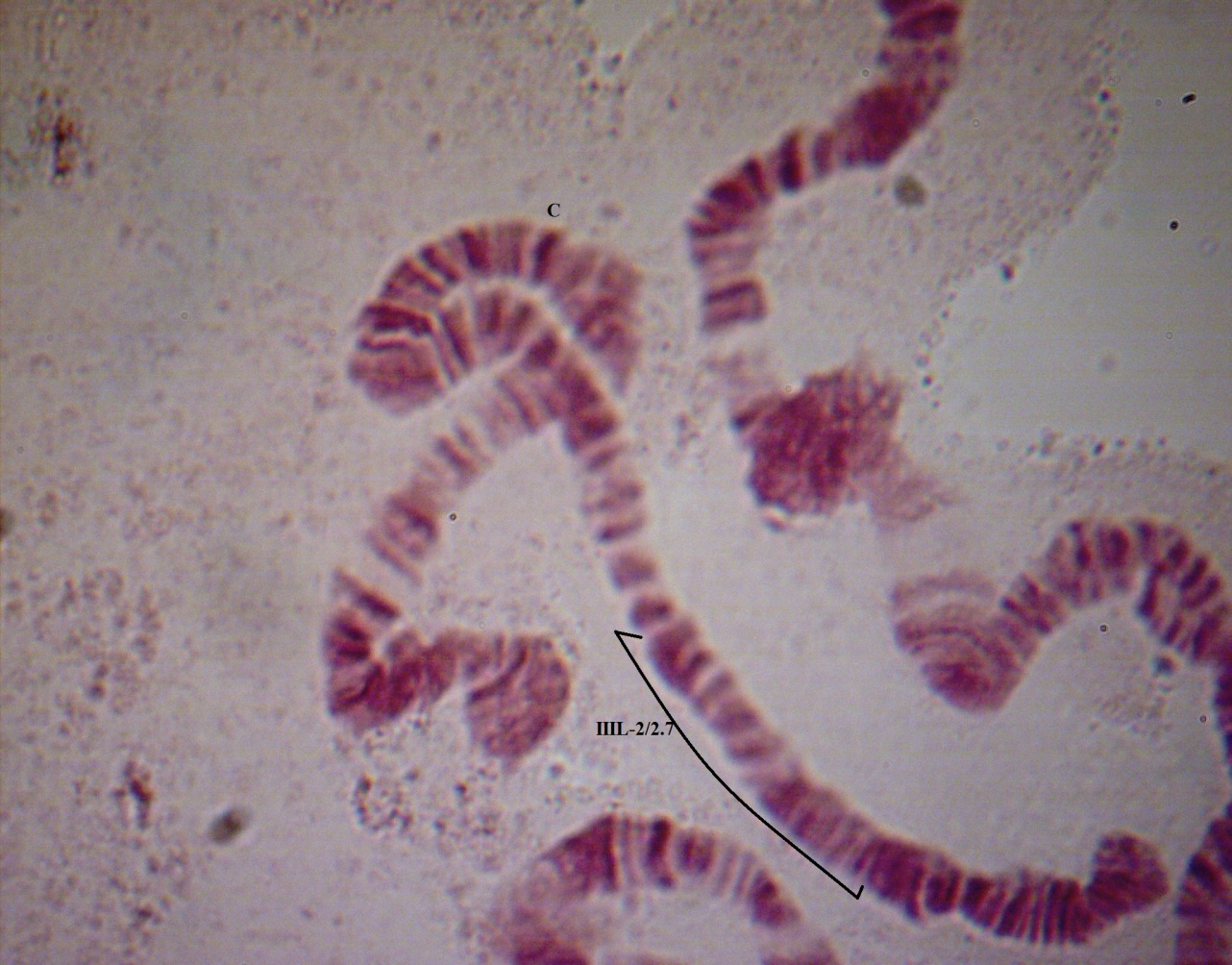
 **S 8:** Chromosome III showing IIIL-2/2.7 homozygous inversions typical of *S. damnosum s.s./S. sirbanum* C = centromere.


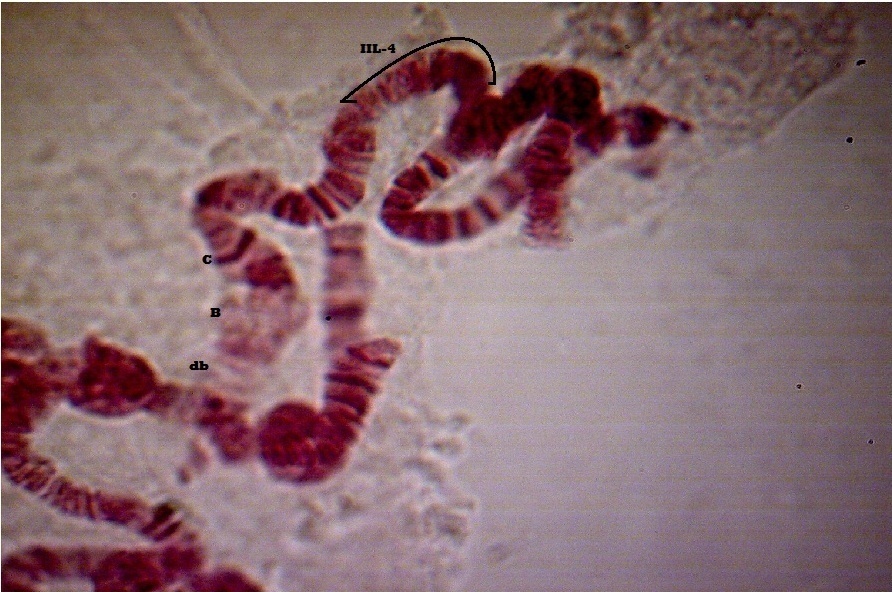
 **S 9:** Chromosome II showing IIL-4 homozygous inversion typical of members of the *S. sanctipauli* s.l. C = centromere, B = balbiani ring, db = double bouble.


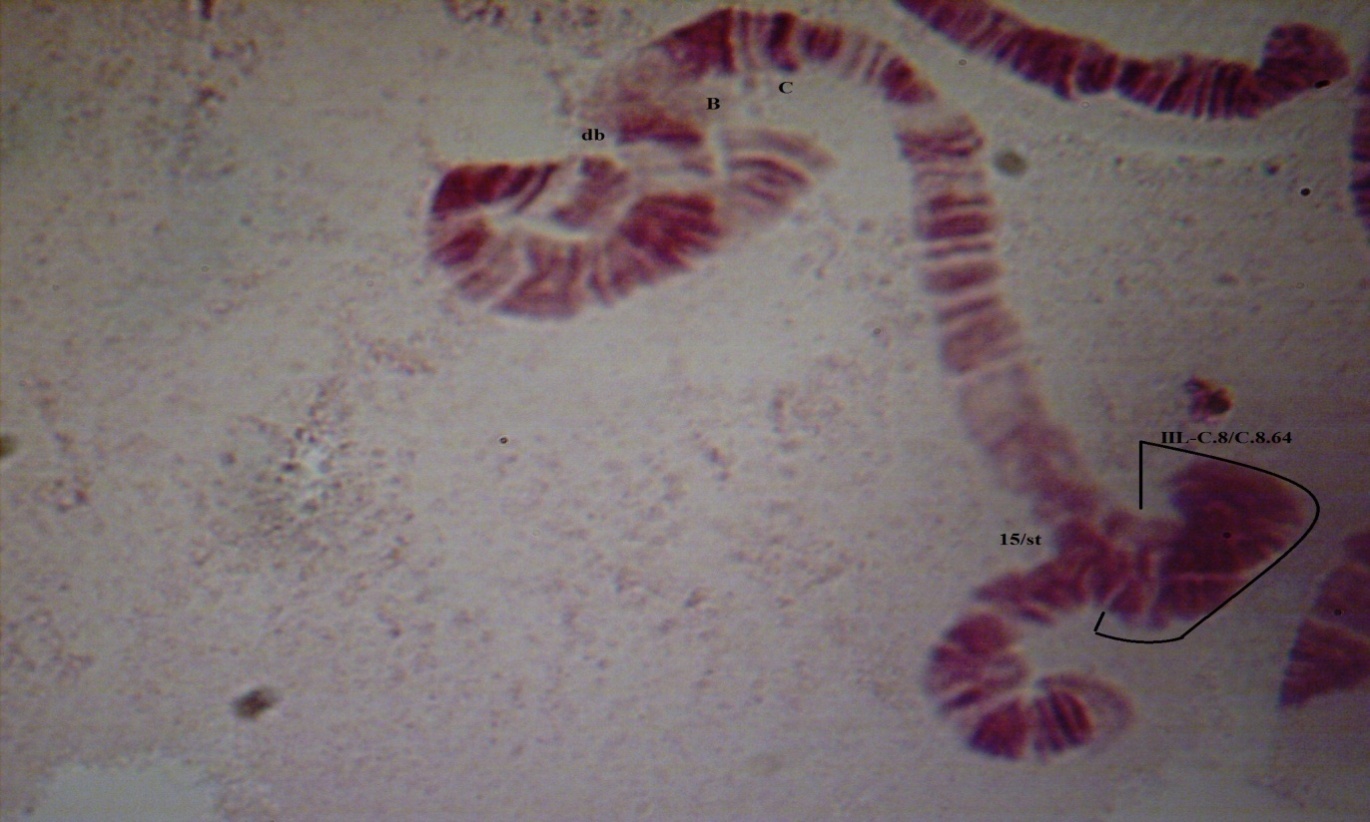
 **S 10:** Chromosome II showing IIL-C.8/C.8.64 and IS/st inversions typical of *S. sirbanum* C = centromere, B = balbiani ring, db = double bouble.
